# *Acinetobacter baumannii* Strains Deficient in the Clp Chaperone-Protease Genes Have Reduced Virulence in a Murine Model of Pneumonia

**DOI:** 10.3390/pathogens10020204

**Published:** 2021-02-13

**Authors:** J Christian Belisario, Hiu Ham Lee, Harshani Luknauth, Nathan W. Rigel, Luis R. Martinez

**Affiliations:** 1 Department of Physical Medicine and Rehabilitation, University of Pennsylvania, Philadelphia, PA 19146, USA; jchristian.belisario@pennmedicine.upenn.edu; 2 Department of Biomedical Sciences, NYIT College of Osteopathic Medicine, New York Institute of Technology, Old Westbury, NY 11568, USA; hlee37@nyit.edu; 3 Department of Biology, Hofstra University, Hempstead, NY 11549, USA; Hluknauth1@pride.hofstra.edu (H.L.); nathan.w.rigel@hofstra.edu (N.W.R.); 4 Department of Oral Biology, University of Florida College of Dentistry, Gainesville, FL 32610, USA

**Keywords:** *A. baumannii*, biofilms, Clp-protease, infection, macrophages, pneumonia

## Abstract

*Acinetobacter baumannii* has emerged as a significant opportunistic Gram-negative pathogen and causative agent of nosocomial pneumonia especially in immunocompromised individuals in intensive care units. Recent advances to understand the contribution and function of *A. baumannii* virulence factors in its pathogenesis have begun to elucidate how this bacterium interacts with immune cells and its interesting mechanisms for multi-antibiotic resistance. Taking advantage of the availability of the *A. baumannii* AB5075 transposon mutant library, we investigated the impact of the *A. baumannii* Clp genes, which encode for a chaperone-protease responsible for the degradation of misfolded proteins, on bacterial virulence in a model of pneumonia using C57BL/6 mice and survival within J774.16 macrophage-like cells. Clp-protease *A. baumannii* mutants exhibit decreased virulence in rodents, high phagocytic cell-mediated killing and reduced biofilm formation. Capsular staining showed evidence of encapsulation in *A. baumannii* AB5075 and Clp-mutant strains. Surprisingly, *clpA* and *clpS* mutants displayed irregular cell morphology, which may be important in the biofilm structural deficiencies observed in these strains. Interestingly, *clpA* showed apical-like growth, proliferation normally observed in filamentous fungi. These findings provide new information regarding *A. baumannii* pathogenesis and may be important for the development of therapies intended at reducing morbidity and mortality associated with this remarkable pathogen.

## 1. Introduction

*Acinetobacter baumannii* is a Gram-negative coccobacillus, motile and biofilm-forming opportunistic pathogen [1]. *A. baumannii* causes a variety of hospital-related infections including ventilator-associated pneumonia and bloodstream, wound and urinary tract infections that can become complicated resulting in meningitis and osteomyelitis [1,2]. In the past two decades, *A. baumannii* has emerged as a major worldwide burden in countries with developed healthcare systems because of its capacity to rapidly develop antibiotic resistance and persist in challenging environments [1,3]. For example, *A. baumannii* strains impervious to carbapenems [4] and amikacin [5] have been frequently isolated from clinical specimens. Carbapenem-resistant *A. baumannii* strains were responsible for approximately 8500 infections in hospitalized patients and 700 deaths in the United States in 2017 [6]. *A. baumannii* can also survive desiccation [1] and during outbreaks has been recovered from various sites in the patients’ environment [7]. The ability of *A. baumannii* to attach to solid surfaces and form biofilms give the bacterium an advantage and ideal niche for its extended prevalence in hospital settings and in various hostile environments [1]. 

Biofilm formation in strains of *A. baumannii* is highly variable, although the most conserved genes associated with this collective phenotype are *csuE*, the proposed tip subunit of the chaperone-usher pili involved in the initial surface attachment [8] and *ompA,* a major cell membrane porin that contributes to adhesion and biofilm development [9], both carried by 81–100% of the isolates. For the biofilm-associated protein (Bap), responsible for biofilm maturation [10] and class A extended β-lactamase blaPER-1 enzyme, associated with increased cell adhesiveness [11], detection is variable ranging from 30–66% to 2–64%, respectively. Moreover, carbapenem-resistant *A. baumannii* is listed by the World Health Organization as the number one critical priority pathogen for which novel therapeutics are urgently required; thus, alternative therapies must be explored to combat infections caused by this microorganism [12]. 

*A. baumannii* is a challenging pathogen to control because to date it has a limited number of well-known virulence factors, many of which are not always present or conserved across all strains [13]. The outer membrane protein A (OmpA) is one of the best characterized *A. baumannii*’s virulence factors and the most abundant outer membrane protein (OMP) found in the bacterium [14]. OmpA causes cytotoxicity in the host cells by translocating into mitochondria [9] or the nucleus [14] resulting in apoptosis and DNA degradation, respectively, of eukaryotic cells. Some *A. baumannii* strains can modify their OM and do not produce either O-antigen or lipopolysaccharide (LPS) but instead they synthesize lipooligosaccharide (LOS) [15]. These OM changes provide the bacterium resistance against immune cells’ elimination [16] and antimicrobial drugs [17]. As an added layer of defense beyond the OM, some strains of *A. baumannii* produce an extracellular capsule. The capsule of *A. baumannii* provides resistance against antimicrobial molecules, ability for phenotypic switching and enhances survival in rodents [18,19]. Likewise, nutrient acquisition, particularly metals such as iron, manganese and zinc, are important for *A. baumannii* survival during infection [13]. In this regard, phospholipases C and D are transcriptionally regulated by the ferric uptake regulator, cause lysis of red blood cells and assist in the uptake of iron [20]. In addition, *A. baumannii* encodes diverse secretion [21,22] and efflux pump [23,24,25] systems necessary for survival in the host and resistance to antibiotics. With the evolution of *A. baumannii* multidrug resistant strains regularly collected in hospitals, especially in intensive care units affecting immunocompromised individuals, more studies investigating the virulence factors associated with this opportunistic pathogen are imperative.

A genome-wide screen analysis identified unique *A. baumannii* virulence factors required for survival in the lungs [26]. The study identified 157 genes necessary for persistence of the microbe in the lung using a pneumonia murine model. Several of the genes identified were already known to be virulence factors; however, many of them are novel. We focused on studying a gene (A1S_0476) in the family of Clp genes that encodes a protease responsible for the degradation of misfolded proteins. The Clp genes are required for proliferation and pathogenicity in other Gram-positive and Gram-negative bacteria such as *Staphylococcus aureus* [27], *Salmonella* Typhimurium [28] and *Yersinia pestis* [29]. Therefore, we investigated the role of several Clp genes on *A. baumannii* pathogenicity by comparing the wild-type and mutant strains from the *A. baumannii* AB5075 transposon mutant library [30]. We hypothesized that *A. baumannii* mutant strains deficient in the Clp chaperone-protease would have reduced pathogenicity in a murine model of pneumonia, survival in J774.16 macrophage-like cells and biofilm formation. Indeed, we found that the Clp chaperone-protease mutants demonstrated diminished virulence, lower survival within macrophages and less biofilm formation than wild-type *A. baumannii* AB5075. Understanding the role of the Clp chaperone-protease genes in *A. baumannii* virulence and pathogenesis might be important to control disease caused by this burdensome pathogen, especially in the hospital setting.

## 2. Results

### 2.1. PCR Analysis Was Performed to Validate the Clp Chaperone-Protease Gene Mutations in A. Baumannii Strain AB5075.

**Figure 1:**
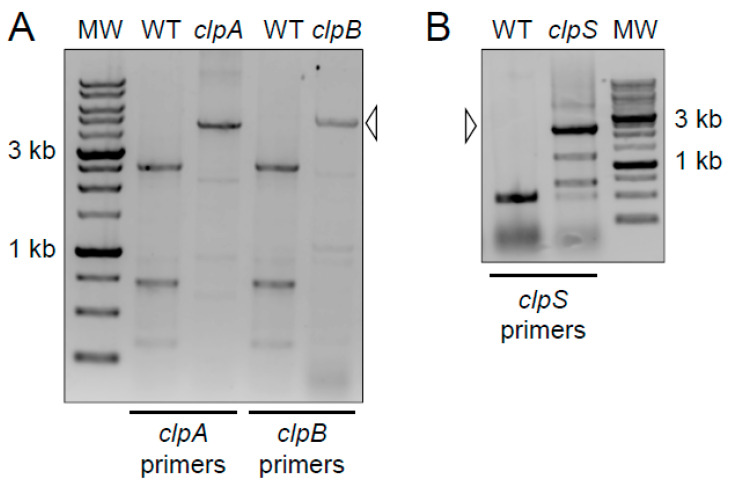
Primers were designed to flank the indicated *clp* gene for amplification by polymerase chain reaction (PCR)**.** The increased size of the products in the mutant lanes (open arrowheads) corresponds to the insertion cassette in the AB5075 mutant library. MW = molecular weight marker. The darker staining bands correspond to 3 kb and 1 kb size standards. The expected PCR products for the wild-type copy of each gene are as follows: (**A**) *clpA* (2560 bp) and *clpB* (2590 bp); (**B**) *clpS* (490 bp). PCR and electrophoresis gel analyses per each genomic DNA isolation (*n* = 2) were performed independently twice and similar results were obtained.

We used polymerase chain reaction (PCR) analysis to confirm the presence of the transposon insertions in the *clpA*, *clpB* and *clpS* genes of the parental *A. baumannii* strain AB5075 (wild-type; Figure 1). Primers were designed to flank each Clp chaperone-protease gene. The expected PCR products for the wild-type copy of *clpA* (2560 bp) and *clpB* (2590 bp) were detected by gel electrophoresis (Figure 1A). In addition, the anticipated PCR product for *clpS* (490 bp) was obtained (Figure 1B). These results verified the integration of the insertion cassette in the AB5075 mutant library and provided validation for each Clp chaperone-protease gene mutant strain utilized in our studies.

### 2.2. Deletion of the Clp Chaperone-Protease Genes Does Not Affect Microbial Growth, Encapsulation and Sensitivity to Oxidative Stress

**Figure 2:**
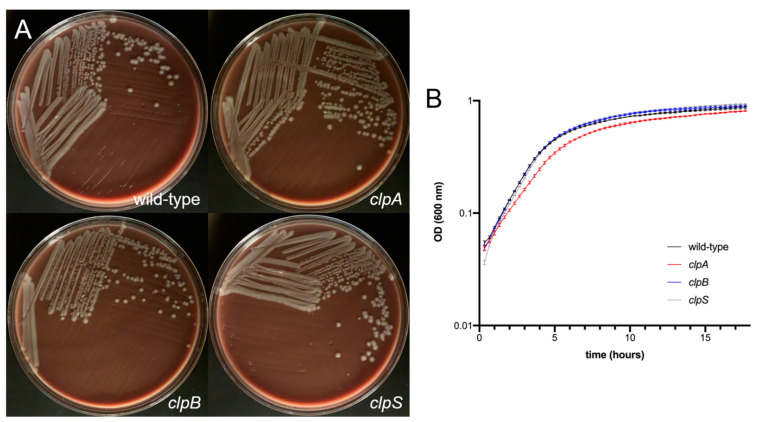
*A. baumannii* AB5075 (wild-type) and *clp* mutant strains demonstrate similar growth rate. (**A**) Colony morphology of wild-type and *clp* mutants of *A. baumannii* AB5075. Overnight cultures of the indicated strains were plated on chocolate agar. Plates were grown for 24 h at 37 °C prior to imaging. (**B**) Growth of wild-type and *clp* mutant strains of *A. baumannii* in 96-well microtiter plates. Five replicate wells were analyzed for each strain. The average optical density (OD) (600 nm) measurement for each time point was then plotted. Error bars indicate standard deviation from the mean. Statistical analyses were calculated by analysis of variance (ANOVA) and adjusted by use of the Tukey’s post-hoc analysis resulting in no significant difference between the strains. Each experiment was performed independently twice and similar results were obtained.

**Figure 3:**
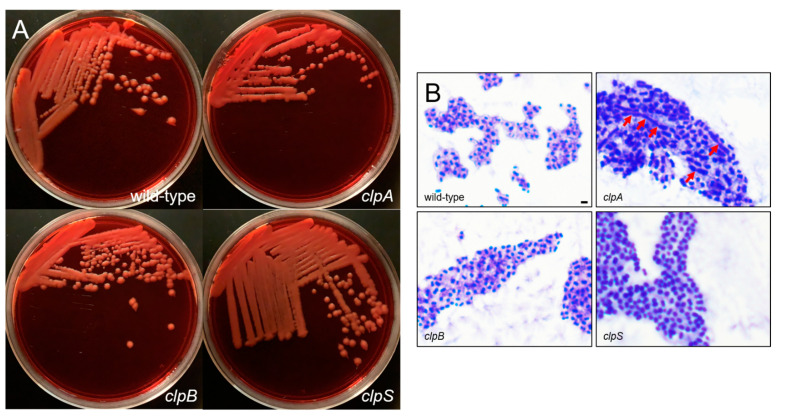
*A. baumannii* AB5075 and *clp* mutants show comparable encapsulation. (**A**) Colony morphology of wild-type and *clp* mutants of *A. baumannii* AB5075. Overnight cultures of the indicated strains were plated on Congo Red agar. Plates were grown for 24 h at 37 °C prior to imaging. (**B**) Cells of the indicated strains were stained with crystal violet and then washed with copper sulfate, prior to qualitative imaging using an Olympus BX51 microscope under the 100X oil immersion lens. The extracellular capsule appears as a pink halo surrounding the purple cells. Red arrows indicate apical-like growth. Scale bar = 5 μm. Each experiment was performed independently twice and similar results were obtained.

**Figure 4:**
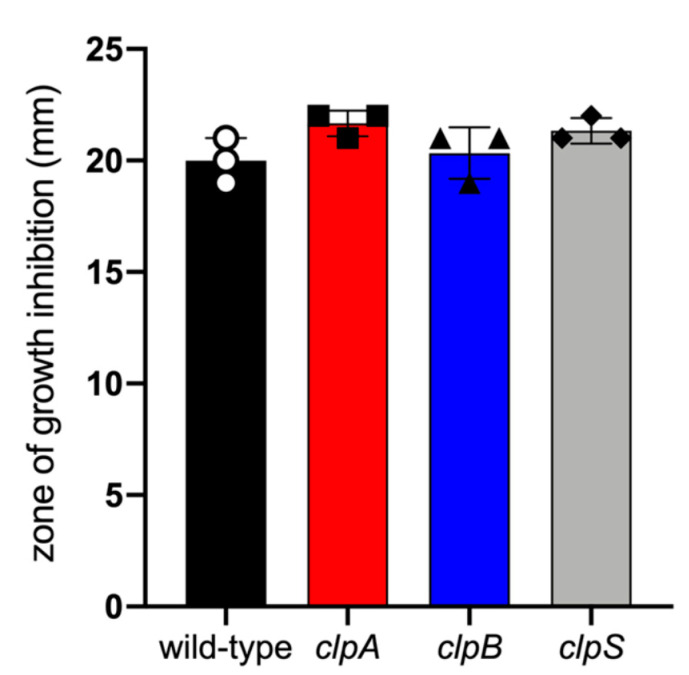
*A. baumannii* AB5075 and *clp* mutant strains had similar sensitivity to oxidative stress. In vitro sensitivity to hydrogen peroxide of *clp* mutants compared to wild-type strain after overnight incubation at 37 °C. Bars indicate the average for each experimental condition and error bars indicate SDs. Each symbol represents an individual replicate (*n* = 3). Statistical analyses were calculated by ANOVA and adjusted by use of the Tukey’s post-hoc analysis resulting in no significant difference between the strains. Each experiment was performed independently twice and similar results were obtained.

Due to the lack of complementation and given that gene deletions may result in aberrant bacterial cell phenotypic and functional defects, we first characterized the impact of Clp chaperone-protease gene deletions on bacterial growth (Figure 2), encapsulation (Figure 3) and sensitivity to oxidative stress (Figure 4).

First, we compared the growth of wild-type *A. baumannii* AB5075, *clpA*, *clpB* and *clpS* mutant strains on chocolate agar incubated for 24 h at 37 °C (Figure 2A). All the strains grew similarly and displayed undistinguishable colony morphology on chocolate agar. Then, we compared the growth of wild-type and the mutant strains on tryptic soy broth incubated at 37 °C under shaking for 18 h. The wild-type and Clp chaperone-protease deficient strains demonstrated similar growth curves (Figure 2B). Although the *clpA* mutant showed a slightly slower growth during the exponential phase (2 to 6 h), this small difference was not statistically significant and *clpA* was able to catch up with the other strains after 18 h.

The binding of Congo Red is associated with the production of bacterial exopolysaccharide such as the capsular material (Figure 3A) [31,32]. The wild-type and the mutant strains were streaked on Congo Red agar and their colony morphology was observed. Each strain demonstrated a creamy and mucoid growth on agar, typically of encapsulated bacteria (Figure 3A). Then, we performed a capsular staining using a combination of crystal violet and copper sulfate (20% *w*/*v*) (Figure 3B). Qualitative assessment demonstrated that all the strains produce a capsule or an extracellular pink material around the cell body. Interestingly, AB5075 (upper left panel, Figure 2B) and *clpB* mutant (lower left panel, Figure 3B) strains displayed similar bacterial cell body (purple) shape and size as well as capsular material distribution. However, the *clpA* (upper right panel, Figure 3B) and *clpS* (lower right panel, Figure 3B) mutants showed larger bacterial cell bodies than the other strains. The *clpA* mutant presented the largest cell body consisting of elongated cells resembling of bacilli connected to other cells in chains via apical-like growth (red arrows; Figure 3B) and surrounded by capsular material. The *clpS* mutant exhibited enlarged coccobacilli shaped cells encircled by moderate amounts of capsular material.

Furthermore, we compared the sensitivity of AB5075 and Clp chaperone-protease deficient strains to oxidative stress by seeding them on Mueller Hinton agar and exposing them to 3% hydrogen peroxide at 37 °C overnight (Figure 4). Similar zones of growth inhibition were documented for all the tested strains indicating similar sensitivity to oxidative stress. These results suggest that mutation of the Clp chaperone-protease genes in *A. baumannii* strain AB5075 do not alter bacterial growth, oxidative stress sensitivity and to some extent encapsulation. Nevertheless, deletion of *clpA* and *clpS* genes alters bacteria cell size and shape and possibly microbial cell separation during cell division.

### 2.3. A. baumannii Requires the Clp Chaperone-Protease Genes for Virulence and to Cause Disease In Vivo

**Figure 5:**
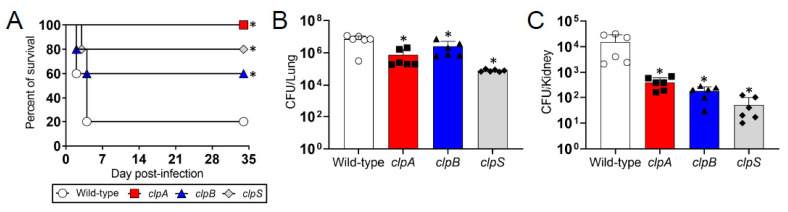
The Clp chaperone-protease genes are necessary for *A. baumannii* virulence and pathogenesis in C57BL/6 mice. (**A**) Survival differences of C57BL/6 mice after intranasal infection with 5 × 10^6^ *A. baumannii* strains AB5075 (wild-type), ClpA, ClpB or ClpS cells (*n* = 5 animals per group). Asterisk denotes *p* value significance (*, *p* < 0.05; lower than wild-type) calculated by log rank (Mantel-Cox) analysis. Bacterial burden (CFU) in lung (**B**) and kidney (**C**) tissue excised from mice sub-lethally infected with 3.75 × 10^6^ cells of *A. baumannii* strains AB5075, *clpA*, *clpB* or *clpS* (*n* = 6 animals per group) after 7 days. Bars and error bars denote the means and standard deviations (SDs), respectively. Each symbol represents an animal. Asterisk indicates *p* value significance (*, *p* < 0.05; lower than wild-type) calculated by ANOVA and adjusted by use of the Tukey’s post-hoc analysis. Each experiment was performed independently twice and similar results were obtained.

We used a murine model of pneumonia to investigate the importance of the Clp chaperone-protease genes in the virulence of *A. baumannii* (Figure 5). We found that C57BL/6 mice infected with a lethal dose of wild-type *A. baumannii* strain AB5075 were killed faster than animals infected with the Clp chaperone-protease deficient strains *clpA*, *clpB* and *clpS* (Figure 5A). For instance, rodents infected with the wild-type bacteria began dying 2 days post-infection and 80% of them were dead 4 days post-infection (Figure 5A). The *clpB* and *clpS* mutants only killed 40 and 20% of the mice, respectively. Interestingly, the *clpA* strain was avirulent given that all the animals infected with this mutant survived for the duration of the experiment (35 days). Mice infected sub-lethally with *A. baumannii* strain AB5075 exhibited significantly higher bacterial burden in pulmonary (Figure 5B) and renal (Figure 5C) tissues than *clpA*, *clpB* and *clpS* mutants (*p* < 0.05). Our findings suggest that Clp genes are important for *A. baumannii* pathogenesis and the fact that the differences in colony forming units (CFU) were ≥1-log 7 days post-infection indicate the important role of this protease in microbial dissemination from the lungs.

### 2.4. The Clp Chaperone-Protease Genes Are Necessary for A. baumannii Survival Inside of J774.16 Macrophage-Like Cells

**Figure 6:**
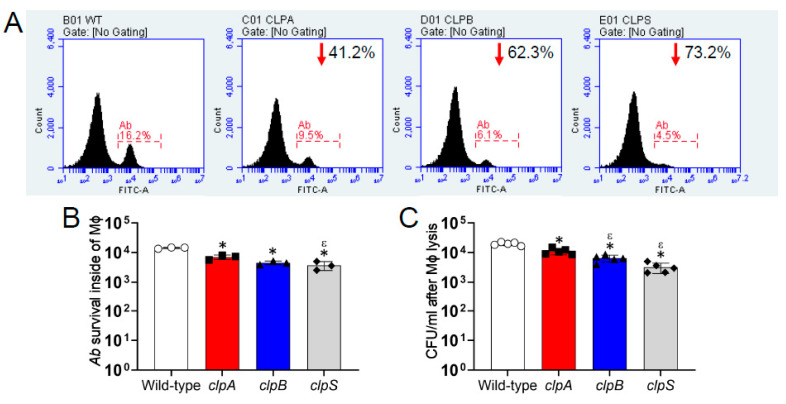
The Clp chaperone-protease genes are important for *A. baumannii* survival within J774.16 macrophage-like cells. (**A**) The killing of *A. baumannii* strains AB5075 (wild-type), *clpA*, *clpB* or *clpS* cells by macrophage-like cells was analyzed by flow cytometry. Representative histograms of FITC-labeled bacteria after lysis of macrophage-like cells are shown. Each plot was generated after 10,000 events were analyzed. The number in the upper right side of the *clp* mutant strains indicates the survival reduction compared to the AB5075 strain. (**B**) Quantification of the number of *A. baumannii* (*Ab*) strain survival inside of macrophages (Mϕ) analyzed by flow cytometry is shown. Each symbol represents an independent experiment. (**C**) CFU determinations after macrophage lysis. Each symbol represents an independent replicate after averaging CFU counts in three separate plates. For panels **B** and **C**, bars indicate the average for each experimental condition and error bars indicate SDs. Asterisk (*; lower than AB5075) and epsilon (ε; lower than *clpA*) indicate *p* value significance (*p* < 0.05) calculated using ANOVA and adjusted by use of the Tukey’s post-hoc analysis. Each experiment was performed independently twice and similar results were obtained.

We assessed the importance of the Clp chaperone-protease genes in the survival of *A. baumannii* after phagocytosis by J774.16 macrophage-like cells using flow cytometry (Figure 6). Relative to wild-type, disruption of the *clpA*, *clpB* and *clpS* genes reduced bacterial survival by 41.2%, 62.3% and 73.2%, respectively (Figure 6A). Quantification of the flow cytometry results indicates that *A. baumannii* strain AB5075 thrive inside J774.16 cells, unlike all three *clp* mutant strains (*p* < 0.05; Figure 6B). The *clpS* mutant displayed significantly lower survival than *clpA* (*p* < 0.05). Although not statistically different, a similar reduced trend was observed when *clpA* and *clpB* were compared. By enumerating CFU after macrophage lysis to validate the results obtained by flow cytometry, we found that the wild-type strain also had higher survival than the Clp chaperone-protease deficient strains inside of the phagocytes (*p* < 0.05; Figure 6C). Additionally, *clpA* showed higher persistence inside of macrophage-like cells than *clpB* and *clpS* mutants (*p* < 0.05; Figure 6C). Our results evince that the Clp chaperone-protease genes are critical for *A. baumannii* survival after engulfment by macrophages.

### 2.5. The Clp Chaperone-Protease Genes Are Crucial for A. baumannii Biofilm Formation In Vitro

**Figure 7:**
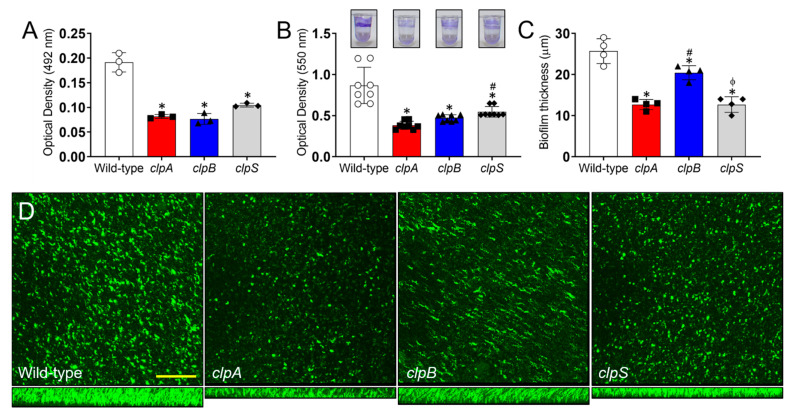
The Clp chaperone-protease genes are essential for *A. baumannii* biofilm formation *in vitro*. *A. baumannii* biofilm formation on polystyrene microtiter plates was evaluated by (**A**) XTT reduction assay and (**B**) crystal violet staining after incubation of AB5075, *clpA*, *clpB* and *clpS* strains (insets) in tryptic soy broth for 48 h at 37 °C. (**C**) The thickness of bacterial biofilms was measured by *Z*-stack reconstruction. For panels **A** to **C**, bars and error bars denote the means and SDs, respectively. Each symbol represents an individual replicate (A, 3 per group; B, 8 per group; C, 4 per group). Asterisk (*), hashtag (#) and phi (ϕ) indicate *p* value significance (*p* < 0.05) calculated by ANOVA and adjusted by use of the Tukey’s post-hoc analysis. * and ϕ denote lower than wild-type and *clpB*, respectively. # indicates higher than *clpA*. (**D**) Confocal microscopy (CM) of mature *A. baumannii* (AB5075, *clpA*, *clpB* and *clpS*) biofilms formed on glass-bottom plates after incubation of the bacteria (green [SYTO 9]) for 48 h at 37 °C. The pictures were taken at a magnification of 63X. Scale bar: 100 µm. Each experiment was performed independently twice and similar results were obtained.

Clp-ATPases are critical for biofilm formation in bacteria [27,33] and this phenotype is important for *A. baumannii* survival as a nosocomial pathogen [7,34]. Thus, we investigated the role of Clp chaperone-protease genes in biofilm formation using the XTT reduction and crystal violet assays and confocal microscopy (Figure 7). *A. baumannii* strain AB5075 exhibited higher metabolic activity and formed more robust biofilms than the Clp chaperone-protease mutant strains (*p* < 0.05; Figure 7A). Using the crystal violet assay, which stains both the cellular components and the exopolymeric matrix of biofilms, confirmed that the wild-type strains adhered strongly to the polystyrene surface of the microtiter plates relative to the Clp chaperone-protease deficient strains (*p* < 0.05; Figure 7B; insets). In addition, the *clpS* mutant evinced higher binding to the polystyrene plate than the *clpA* strain (*p* < 0.05). We used confocal microscopy to study the differences in biofilm architecture and depth between wild-type and Clp chaperone-protease mutant strains (Figure 7C,D). *A. baumannii* strain AB5075 displayed the thickest biofilms (*p* < 0.05; Figure 7C). Interestingly, the *clpB* strain produced deeper biofilms than *clpA* and *clpS* strains (*p* < 0.05; Figure 7C,D), both which had similar biofilm thickness when compared to each other. Furthermore, all the strains showcased homogeneous bacterial cell distribution throughout the field, although the wild-type strain biofilm structures were comprised of the largest bacterial aggregates (Figure 7D). Taken together, our data demonstrate that the Clp chaperone-protease genes are essential to form robust mature biofilms, which is an important attribute for maximizing microbial survival during infection and nature.

## 3. Discussion

We investigated the importance of the Clp chaperone-protease gene expression in *A. baumannii* pulmonary infection. Our data show that the Clp chaperone-protease genes are required for the bacterium to cause disease and thrive within the mammalian host. Like what has been observed in other bacterial pathogens [35,36,37], we have uncovered several specific roles for the *A. baumannii* Clp chaperone-protease system, including intracellular survival and biofilm formation. We confirmed that the defects observed in *A. baumannii clp* mutants after interaction with phagocytes or biofilm formation are not caused by aberrant growth phenotypes under standard laboratory conditions. *A. baumannii* AB5075 and *clp* mutant strains grow at a similar rate and show alike colony morphology. Additionally, we confirmed the validity of the Clp chaperone-protease mutant strains by checking the integration of the insertion cassette in the *A. baumannii* AB5075 mutant library by PCR. Despite the fact, we compared the resistance of *clp* mutant and wild-type strains to hydrogen peroxide, our simple test must be interpreted as a verification of growth similarities between these strains and not as a conclusive result suggesting that *clp* mutants are resistant to oxidative stress. The later possibility needs to be further investigated using more detailed set of experiments.

In bacteria, the ClpP protease is paired with a variety of chaperones to enable regulated proteolysis [38,39]. These chaperones, like ClpA, ClpB and ClpX, are part of the ATPases Associated with diverse cellular Activities (AAA+) superfamily, which are proteolytic machines that degrade damaged and unneeded proteins in all domains of life [40]. Powered by ATP hydrolysis, these factors unfold substrates that are destined for degradation via ClpP. Adaptor proteins like ClpS bind to and modulate the activity of the chaperone-protease complex. This modularity increases the complexity of post-translational regulation employed by bacteria, which is particularly advantageous for pathogens who find themselves needing to rapidly adapt to the challenges of dealing with the defenses of the human immune system. By targeting transcription factors for proteolysis, bacteria can modulate their gene expression profiles in response to new environmental stimuli. Several studies have demonstrated that the avirulent phenotypes associated with protease-deficient mutants are due in large part to such aberrant gene regulation [41,42,43]. Interestingly, we found that the *clpA* and *clpS* mutant strains exhibit formidable phenotypic changes including alterations to cell size, shape and especially in *clpA*, a possible defect in bacterial separation during replication. Although further experimentation is required to confirm this result, we observed apical growth in the *clpA* mutant, like filamentous fungi during hyphal formation. Filamentous growth has been previously reported in a *Bacillus subtilis clpP* mutant and is partially caused by defects in the first step of peptidoglycan biosynthesis [44]. Despite evidence suggesting the impact of the Clp-chaperone protease system in cell wall metabolism and cell division in Gram-positive and Gram-negative bacteria, the molecular pathways possibly involved remain an unexplored and potential area of research [45].

In addition to ClpA, ClpB and ClpS, the genome of *A. baumannii* AB5075 is predicted to encode several common protease-chaperones including ClpP, ClpU, Lon, Ctp and Prc [18,30,46]. While these factors have been well-described in other organisms, the function of these proteins, particularly during infection, are poorly understood in *A. baumannii*. For example, Lon modulates biofilm formation and cell envelope integrity, though the specific mechanism through which Lon operates remains unclear [47]. Mutants lacking the C-terminal protease Ctp are attenuated in both cell culture and whole-animal infection assays [46]. Both, Lon and Ctp, are important in protecting *A. baumannii* against environmental stress, suggesting the importance of chaperone-proteases in pathogenesis or antibiotic resistance. Combine with the finding we have presented here, there is a growing body of evidence implicating chaperone-proteases as key virulence determinants in *A. baumannii*.

We found that ClpA and ClpB are necessary for survival of *A. baumannii* within macrophages. Given the involvement of chaperone-proteases in bacterial survival during stressful conditions, it is conceivable that ClpA and ClpB protect *A. baumannii* against the acidity of the phagolysosome and oxidative burst of macrophages. ClpB facilitates the survival of *Listeria monocytogenes* and *Mycobacterium tuberculosis* in macrophages, indicating a potential role of ClpB in bacterial persistence [48,49]. Deletion of *clpA* and *clpB* in *S.* Typhimurium results in increased susceptibility to macrophage phagocytosis and oxidative stress [50]. In agreement with the results in *S.* Typhimurium, the loss of *clpB* by *A. baumannii* makes the microbe more susceptible to killing by macrophages than did the deletion of *clpA*. Similarly, the expression of the *Ehrlichia chafeensis clpB* gene is upregulated following infection of macrophages [51], indicating an important role of ClpB protein under oxidative stress. Moreover, recent reports implicate ClpB together with Type VI secretion system in delivering effectors for phagosomal escape of *Francisella tularensis* [52] and biofilm formation in *M. tuberculosis* [49]. Although the relationship between *A. baumannii* chaperone-proteases and a secretion system in phagocytic cell exocytosis requires future studies yet for elucidation, other chaperones such as GroEL1 [53], DnaK [54] and DnaJ [55] has been shown regulatory involvement in biofilm formation by multiple bacteria. Interestingly, this is the first report that demonstrate that ClpS is also needed by *A. baumannii* for intracellular survival. Therefore, the chaperone-protease genes are critical for the survival of the *A. baumannii* inside of macrophages and their colonization of pulmonary tissue during pneumonia.

Our studies suggest that the Clp chaperone-protease genes, particularly *clpA* and *clpS*, are critical for *A. baumannii* biofilm formation, an important feature utilized by the bacterium to thrive in the clinical setting. Confocal microscopy demonstrated that *clpA* and *clpS* disruption in *A. baumannii* substantially impairs the biofilm architecture likely due to the cell morphological modifications reported in this report. Differences in *A. baumannii* strain AB5075 cell shape, biofilm formation, motility, antibiotic resistance and virulence have been documented previously in colonies with opaque and translucent phenotypes [56]. To a lesser effect, disruption of *clpB* diminished *A. baumannii* biofilm thickness. As noted in *M. tuberculosis*, this gene provides mycobacteria with the ability to persist in latency-like conditions such as prolonged hypoxia and nutrient-starvation [49], which might be important for *A. baumannii* to survive lengthy desiccation periods in the clinical environment. Additionally, *A. baumannii* AB5075 polysaccharide capsule might suffer structural modifications associated to the *clp* mutations and this can have implications in biofilm formation. For instance, an opaque AB5075 strain with a thicker capsule has more resistance to disinfectants, host antimicrobial molecules and desiccation [57]. The possibility of Clp-mediated disruption of microbial cell encapsulation and its effect on *A. baumannii* biofilm formation warrants future detailed investigations that are out of the scope of this study. Accumulating data suggests that Clp proteases and chaperones are necessary for biofilm formation regulation in both Gram-positive and Gram-negative bacteria such as *S. aureus* [27] and *Porphyromonas gingivalis* [33], respectively.

## 4. Conclusions

This study shows that chaperone-protease functions as an essential stress regulator for *A. baumannii* as it helps the pathogen in adapting to numerous stressful conditions and imparts it survival advantage in macrophages and pneumonia. Even though additional studies are needed, chaperone-proteases appear to have a critical role in broad arrays of processes associated with bacterial antibiotic resistance, motility, biofilm formation, virulence and stress adaptation. Further research is also required to fully understand how ATP-dependent proteases can act as global regulators inside the bacterial cell, as well as their potential as novel antimicrobial targets. For example, *A. baumannii* strain AB5075 capsule is important for survival in a murine model of pneumonia [57]. The role of the Clp-chaperone protease system on capsular production should be investigated because it can have profound implications in biofilm formation, environmental survival and pathogenesis. This is ultimately relevant to the clinic, particularly for the treatment of *A. baumannii* infection, where the bacterium causes acute pulmonary disease that can lead to mortality mainly in immunocompromised individuals. Since *A. baumannii* is difficult to control with commonly available antibiotics, it is imperative to find novel therapeutic alternatives. Chaperone-proteases such as ClpP, ClpB and ClpS are plausible candidates for the development of new therapeutics to combat *A. baumannii* due the potency demonstrated against other similar pathogens.

## 5. Materials and Methods

### 5.1. Acinetobacter baumannii

*A. baumannii* strain AB5075, a clinical isolate from a human patient with osteomyelitis of the tibia in Maryland, USA., was chosen for this study because it has been sequenced, its multi-drug resistant profile and increased virulence in animal models [58]. AB5075 transposon insertion mutants for *clpA* (tnab1_kr130917p01q124), *clpB* (tnab1_kr121205p01q133) and *clpS* (tnab1_kr121212p02q133) were obtained from the sequence-verified transposon mutant library for AB5075 [30]. The strains were stored at −80 °C in brain heart infusion (Becton Dickinson [BD]) broth with 40% glycerol until use. Strains were subsequently propagated in the laboratory by growth in tryptic soy broth (MP Biomedicals) overnight at 37 °C using an orbital shaker incubator set at 150 rpm. Where indicated, strains were streaked for isolation on chocolate agar (Hardy Diagnostics) or Congo Red agar and incubated at 37 °C for 24 h. Per liter, Congo Red agar was composed of 37 g brain heart infusion broth, 5 g sucrose (Sigma), 10 g agar (BD) and 0.8 g Congo Red (VWR). Growth of broth cultures was monitored by measuring the optical density (OD) at 600 nm using a microtiter plate reader (BioTek).

### 5.2. PCR Analysis of clp Genes

To confirm transposon insertion, each *clp* gene was amplified by colony PCR and the resulting products were analyzed by agarose gel electrophoresis. PCR reactions were assembled using the Q5 high-fidelity DNA polymerase following the manufacturer’s instructions (Qiagen), using the following primers: *clpA* (5′- TGCAGTGGTATTATTGAATG-3′ and 5′-TCGGGTTATAGAAAGACTAT-3′), *clpB* (5′-CGAGTTTTACTCGCAGTATT-3′ and 5′-TGCGCTTATATCTTCAAATC-3) and *clpS* (5′-GCTGAAGCAAATTCACAATA-3′ and 5′-CCAAACGTAATGATACTTCT-3′).

### 5.3. Growth Curves

Overnight cultures of each strain were diluted 1:100 in fresh tryptic soy broth into the wells of a 96-well plate. The plate was then incubated at 37 °C with shaking in a SpectraMax i3x plate reader. OD measurements at 600 nm were taken every 20 min for 18 h. Four replicates wells were seeded for each strain. The average OD measurement for each time point was then plotted.

### 5.4. Capsule Staining

Overnight cultures of each strain were used to prepare smears on glass slides. After spotting 10 µL of culture onto a slide and allowing it to air dry, the smears were stained using crystal violet. The excess dye was washed off and counter-stained using copper sulfate (20% *w*/*v*). Slides were visualized using the 100X oil immersion objective on an Olympus BX51 microscope.

### 5.5. Sensitivity to Hydrogen Peroxide

Mueller Hinton agar plates were inoculated with overnight cultures of each strain by soaking a sterile cotton swab in bacterial culture and spreading the swab on the agar surface. Next, 5 µL of 3% hydrogen peroxide was spotted on the agar surface and allowed to air dry. Plates were then inverted and incubated at 37 °C overnight. The next day, zones of growth inhibition were measured around the hydrogen peroxide spots.

### 5.6. Bacterial Infection

All animal studies were conducted according to the experimental practices and standards approved by the Institutional Animal Care and Use Committee at NYIT College of Osteopathic Medicine (Protocol #: 2016-LRM-01). The IACUC at NYIT College of Osteopathic Medicine approved this study. To assess survival rates, female C57BL/6 mice (age, 6–8 weeks; Charles Rivers) were anesthetized [100 mg kg^−1^ ketamine (Keta-set; Covetrus), 10 mg kg^−1^ xylazine (Anased; Covetrus)] and intranasally infected with 5 × 10^6^ bacteria for each *A. baumannii* strain. For bacterial burden studies, a sublethal infection was performed by intranasal inoculation of 3.75 × 10^6^ *A. baumannii* cells.

### 5.7. CFU Count Determinations in Tissues

At day 7 post-infection, mice were euthanized with 30–70% CO_2_ in a chamber. Mouse pulmonary and renal tissues (left lung and kidney) were excised and homogenized in sterile phosphate-buffered saline (PBS). Serial dilutions of homogenates were made; a 100 µL suspension of each sample was then plated on tryptic soy agar (MP Biomedicals) plates and incubated at 37 °C for 24 h. Quantification of viable bacterial cells was determined by CFU counts and the results were normalized per organ.

### 5.8. J774.16 Macrophage-Like Cells

The J774.16 macrophage cell line originated from a murine reticulum cell sarcoma and has been extensively used to study microbe–macrophage interactions (American Type Culture Collection). The J774.16 cells were stored at −80 °C prior to use. The J774.16 cells were suspended in Dulbecco’s modified Eagle’s medium with 10% heat-inactivated fetal calf serum (Biotechne), 10% NCTC-109 (Gibco) and 1% non-essential amino acids (Corning), passaged three to four times and grown as confluent monolayers on tissue culture plates prior to each experiment.

### 5.9. Phagocytosis and Killing Assay

Phagocytosis was determined by flow cytometry analysis. J774.16 macrophages (10^6^ cells) were incubated on 96-well plates (200 µL per well; Corning) for 24 h at 37 °C and 5% CO_2_. *A. baumannii* cells labeled with fluorescein isothiocyanate (FITC; Molecular Probes) were incubated with 25% mouse serum (Sigma) for 30 min to allow complement proteins to opsonize the bacterial cells. Bacterial cells were rinsed with culture medium and then 10^7^ bacteria were added and incubated with J774.16 cells for 2 h to allow phagocytosis at 37 °C and 5% CO_2_. To get rid of extracellular bacteria, each well was gently rinsed twice with 200 µL of fresh culture medium followed by the addition of 200 µl of culture medium supplemented with 2 µg/mL of amikacin (MP Biomedicals) and incubation at 37 °C and 5% CO_2_. Quantification of viable bacteria was determined at 18 h by flow cytometry analysis or measuring CFU after macrophages had been lysed by forcibly passing the culture through a 27-gauge needle 5–7 times [59,60]. For flow cytometry analysis, bacteria were incubated with propidium iodide for 30 min. Then, samples (10,000 events per sample) were processed on an LSRII flow cytometer (BD) and the results were analyzed using FlowJo software. The percentage of infected macrophages was 95% for each condition. Samples containing only macrophages or bacteria were used as controls to differentiate between infected and uninfected macrophages in the population. For CFU counts, five microtiter wells per experimental condition were used. For each well, serial dilutions were plated in triplicate onto tryptic soy agar plates, which were incubated at 37 °C for 24 h.

### 5.10. Biofilm Formation

For each strain, 100 μL of a suspension with 10^6^ bacterial cells in tryptic soy broth medium was added into individual wells of polystyrene 96-well plates or poly-d-lysine 35-mm glass-bottom plates (MatTek) and the plates were incubated at 37 °C without shaking. The biofilms could form for 24 h. Biofilms were rinsed 3 times with phosphate-buffered saline (PBS) to remove nonadherent bacteria and 100 μL of fresh medium was added.

### 5.11. Measurement of Biofilm Metabolic Activity by XTT Reduction Assay

A semiquantitative measurement of *A. baumannii* biofilm formation was obtained from the 2, 3-bis (2-methoxy-4-nitro-5-sulfophenyl)-5-[(phenylamino) carbonyl]-2H-tetrazolium-hydroxide (XTT; Sigma) reduction assay [34]. Aliquots of 50 µL of XTT salt solution (1 mg mL^−1^ in PBS) and 4 µL of menadione solution (1 mM in acetone; Sigma) were added to each well of a microtiter plate. Microtiter plates were then incubated at 37 °C for 5 h. The electron transport system in the cellular membrane of live bacteria reduces the XTT tetrazolium salt to XTT formazan, resulting in a colorimetric change, which was measured in a microtiter plate reader at 492 nm. Microtiter wells containing only tryptic soy broth without *A. baumannii* bacterial cells were used as negative controls.

### 5.12. Crystal Violet Assay

*A. baumannii* biofilm formation on polystyrene microtiter plates was measured by crystal violet staining as described elsewhere [34]. Each well of a 96-well plate containing mature bacterial biofilms was stained with 100 µL of a 0.1% crystal violet solution (ThermoFisher) for 15 min. Then, each well was rinsed three times with distilled water (dH_2_O) before a suspension of 30% acetic acid in dH_2_O was added to each well containing biofilms to solubilize the crystal violet and the plate was incubated at room temperature for 15 min. Finally, solubilized crystal violet was measured in a microtiter reader at 550 nm using 30% acetic acid in dH_2_O as negative control.

### 5.13. Confocal Microscopy

The structural integrity of *A. baumannii* biofilms was examined using the Live/Dead biofilm viability kit (Invitrogen) and confocal microscopy. Briefly, *A. baumannii* biofilms were grown for 24 h in 35-mm glass-bottom culture dishes, rinsed three times with dH_2_O and incubated for 30 min at room temperature in 2 mL of dH_2_O containing the fluorescent stain SYTO9 (6 μL; Invitrogen), with protection from light. SYTO9 (excitation wavelength, 500 nm; emission wavelength, 535 nm) labels bacteria within the biofilm. The dishes were then rinsed three times with dH_2_O to remove excess stain. Microscopic examinations of biofilms formed in culture plates were performed with confocal microscopy, using an inverted Leica TCS SP5 confocal laser scanning microscope (Leica).

### 5.14. Statistical Analysis

All data were subjected to statistical analysis using GraphPad Prism 9.0 (GraphPad Software). Differences in survival rates were analyzed by the log-rank test (Mantel-Cox). *p* values for multiple comparisons were calculated by analysis of variance (ANOVA) and adjusted using the Tukey’s multiple comparison test. *p* values of <0.05 were considered significant.

## Data Availability

All raw data is available and provided upon request.
